# Dual roles for ubiquitination in the processing of sperm organelles after fertilization

**DOI:** 10.1186/1471-213X-14-6

**Published:** 2014-02-15

**Authors:** Connie Hajjar, Katherine M Sampuda, Lynn Boyd

**Affiliations:** 1 University of Alabama in Huntsville, Huntsville, AL, USA; 2 Middle Tennessee State University, Murfreesboro, TN, USA

## Abstract

**Background:**

The process of fertilization involves a cell fusion event between the sperm and oocyte. Although sperm contain mitochondria when they fuse with the oocyte, paternal mitochondrial genomes do not persist in offspring and, thus, mitochondrial inheritance is maternal in most animals. Recent evidence suggests that paternal mitochondria may be eliminated via autophagy after fertilization. In *C. elegans,* sperm-specific organelles called membraneous organelles (MO) cluster together with paternal mitochondria immediately after fertilization. These MOs but not the mitochondria become polyubiquitinated and associated with proteasomes. The current model for the elimination of paternal mitochondria in *C. elegans* is that ubiquitination of the MOs induces the formation of autophagosomes which also capture the mitochondria and cause their degradation.

**Results:**

Sperm-derived mitochondria and MOs show a sharp decrease in number during the time between sperm-oocyte fusion and the onset of mitosis. During this time, paternal mitochondria remain closely clustered with the MOs. Two types of polyubiquitin chains are observed on the MOs: K48-linked ubiquitin chains which are known to lead to proteasomal degradation and K63-linked ubiquitin chains which have been linked to autophagy. K48-linked ubiquitin chains and proteasomes show up on MOs very soon after sperm-oocyte fusion. These are present on MOs for only a short period of time. Maternal proteasomes localize to MOs and sperm proteasomes localize to structures that are at the periphery of the MO cluster suggesting that these two proteasome populations may have different roles in degrading paternal material. K63-linked ubiquitin chains appear on MOs early and remain throughout the first several cell divisions.

**Conclusions:**

Since there are two different types of polyubiquitin chains associated with sperm organelles and their timing differs, it suggests that ubiquitin has two or more roles in the processing of sperm components after fertilization. The K63 chains potentially provide a signal for autophagy of paternal organelles, whereas the K48 chains and proteasomes may be involved in degradation of specific proteins.

## Background

Most animals inherit their mitochondrial genomes exclusively from their maternal parent. The genetics of this inheritance pattern has been well described, however the cell biology of this problem has not been fully elucidated. Strict maternal inheritance of mitochondria is inconsistent with the observation that sperm cells contain a significant number of mitochondria. When sperm and oocytes fuse during the process of fertilization, these sperm mitochondria become incorporated into the cytoplasm of the oocyte. The mechanism by which these paternal mitochondria are excluded from the mitochondrial pool of the new individual is not completely clear. Studies in *C. elegans* indicate that ubiquitination and subsequent autophagy may account for the removal of these sperm-derived mitochondria [1-3].

Ubiquitin is involved in a number of cellular processes including protein degradation, vesicle sorting, DNA repair, autophagy and transcription [4]. Ubiquitin is a small protein that is attached post-translationally to other proteins via a conserved enzymatic pathway. Ubiquitin is linked to proteins via an isopeptide bond between the C-terminal glycine of ubiquitin and the amino group on the side chain of a lysine residue on the target protein. In some cases a single ubiquitin is added onto a protein. Other times, a chain of ubiquitins is generated by linking ubiquitin peptides to each other via isopeptide linkages between a lysine residue in ubiquitin and the C-terminal glycine of the other ubiquitin. In this way, long unbranched chains of ubiquitin are added on to proteins. These chains come in seven different varieties depending on which lysine of ubiquitin is used for the attachment. The most common chains detected in cell extracts are chains formed via linkage to lysine 48 (K48) or lysine 63 (K63) [5]. It has been shown that K48 chains of 4 ubiquitins or longer can be recognized by the proteasome [6]. K63 chains are associated with NF-κB signaling and are also found on structures that eventually become autophagocytized [7,8]. Ubiquitin binding domains that have preference for one type of chain (either K48 or K63) are believed to be responsible for the different effects of the two chain types [9]. In the case of autophagy, proteins such as p63 or NBR1 are known to bind K63 chains with a UBA domain and also bind to autophagy membranes via a LIR domain [10].

The ubiquitin pathway may be involved in targeting sperm organelles for degradation. There is evidence that this is a selective process. Research in mice has shown that sperm mitochondria are specifically recognized by the oocyte since mitochondria from liver are not eliminated when injected into oocytes [11]. Also, interspecies sperm injections do not result in elimination of sperm mitochondria whereas intraspecies injections do, again suggesting that a species specific molecule serves to target sperm mitochondria for elimination after fertilization [12]. Reports from mammalian systems have shown that sperm mitochondria are associated with ubiquitin after fertilization [13,14]. This is consistent with the nematode results and suggests that autophagy may also be involved in eliminating these paternal mitochondria.

We have studied the phenomenon of paternal mitochondrial elimination in the nematode, *C. elegans.* This species shows maternal inheritance of mitochondria [15]. The association of ubiquitin with eliminated sperm organelles suggests that the mechanism for elimination of sperm organelles might be similar in mammals and *C. elegans. C. elegans* spermatozoa harbor a highly folded organelle termed the membraneous organelle (MO) whose function is not known. The MOs undergo a morphological change when spermatids mature into spermatozoa. At this time, the MOs release major sperm protein intracellularly and many of the MOs fuse with the plasma membrane [16]. Electron micrographs of sperm cells suggest that the MOs and the mitochondria are the only cytoplasmic organelles present in sperm cells [17].

The current study investigates the timing of paternal organelle ubiquitination and elimination in *C. elegans* embryos. Our results confirm that sperm mitochondria enter the oocyte upon fusion. These mitochondria are closely associated with the MOs initially. The MOs quickly become ubiquitinated. The number of both MOs and mitochondria decrease rapidly during the completion of maternal meiosis. The sperm mitochondria and MOs are initially clustered together after fertilization. However, after the completion of maternal meiosis, during the process of cytoplasmic streaming and initiation of the first mitotic cycle, these organelles begin to disperse in the zygotic cytoplasm. Staining with linkage-specific anti-ubiquitin antibodies reveals that both K48 and K63 linked chains are present on MOs in embryos just after fertilization. K48-linked ubiquitin chains are present for only a short time, whereas K63-linked ubiquitin chains persist until the MOs are no longer present. Proteasomes are also associated with the sperm MOs after fertilization and our results indicate that these are maternally derived rather than sperm supplied proteasomes.

## Results

### Elimination of sperm organelles occurs mostly prior to the first mitosis

**Figure 1:**
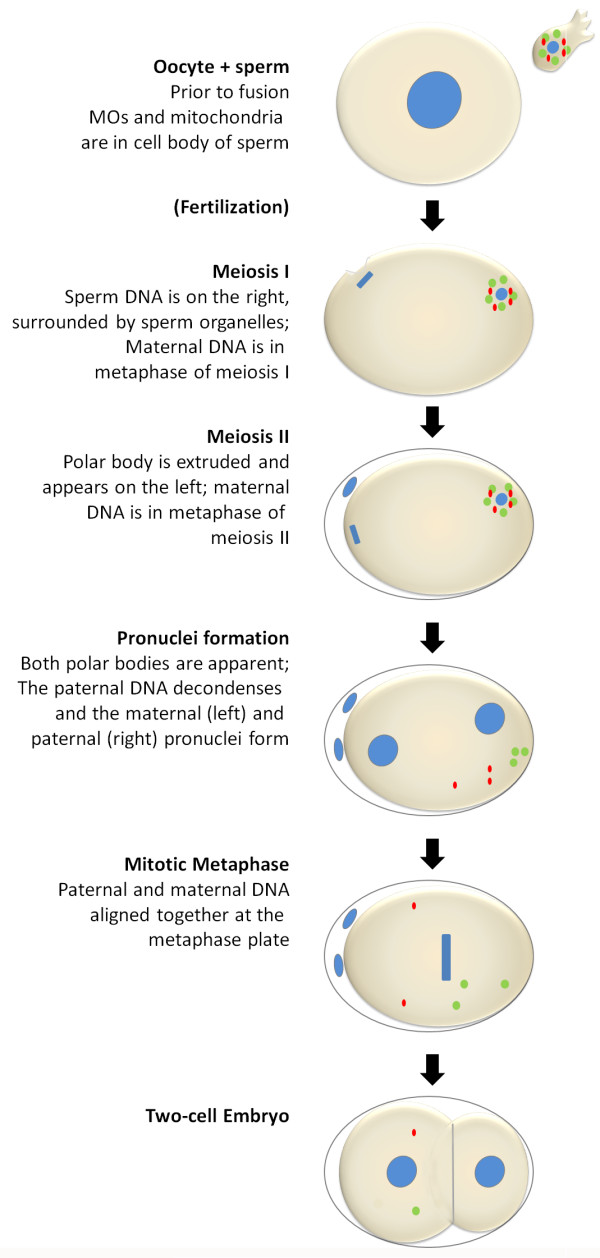
**Fertilization in *C. elegans.*** Oocytes are arrested in prophase of meiosis I. Fertilization occurs in the spermatheca of the adult hermaphrodite. A single, mature spermatozoon fuses with the oocyte. After fusion, the maternal genome completes meiosis I resulting in the formation of the first polar body. Meiosis II ensues immediately and leads to the formation of the second polar body. During this time, the sperm DNA remains in a highly condensed state. The sperm organelles stay closely associated with the sperm DNA. Immediately following the completion of meiosis II, the sperm DNA decondenses and the maternal and paternal pronuclei are formed. The maternal pronucleus migrates to the sperm pronucleus and upon joining, the first mitotic cell division commences. This entire process occurs within the first half hour after fertilization. DNA is shown in blue, MOs are green, and mitochondria are red.

In *C. elegans* oocytes are arrested in prophase of meiosis I until fertilization. In response to secreted signals from sperm, a mature oocyte moves into the spermatheca where fertilization occurs [18]. The location where the sperm fusion occurs on the oocyte determines the posterior end of the embryo [19]. After fusion, the maternal genome completes meiosis and two polar bodies are formed (diagram in Figure 1). The oocyte acquires sperm chromatin as well as cytoplasmic material such as mitochondria, membraneous organelles (MO), and major sperm protein (MSP) during the process of fusion. Some of these sperm-derived cytoplasmic components are degraded via autophagy [1].

**Figure 2:**
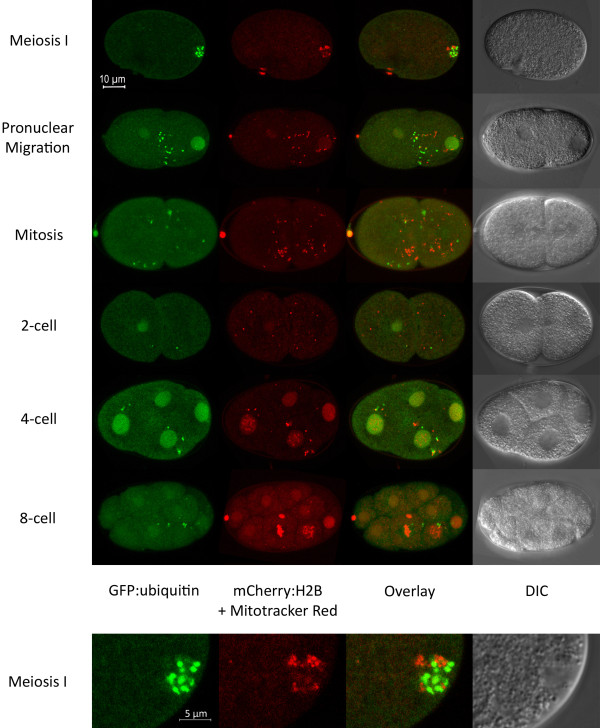
**Paternal mitochondria and membraneous organelles can be visualized after fertilization.** Mitotracker labelled males were mated to hermaphrodites expressing GFP::Ub. Embryos were imaged via confocal microscopy after fertilization and Z stacks were collected. Maximum projection images are shown. The ubiquitinated MOs (green) are closely associated with the sperm mitochondria (red) until the completion of maternal meiosis. As the embryo enters mitosis, the MOs and mitochondria become dispersed.

**Figure 3:**
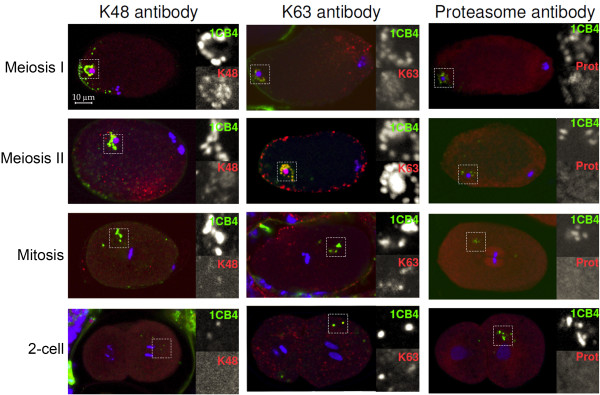
**Ubiquitin and proteasome localization in early embryos.** Chain linkage specific anti-ubiquitin antibodies and an antibody to a subunit of the 19S proteasome were used to stain embryos (red). The embryos were also stained with the 1CB4 antibody which recognizes the MO organelles from sperm (green). DAPI was used to visualize the DNA (blue). All embryos are oriented with anterior end at the right. The whole embryo is shown as well at an enlargement of the area of the MOs. MOs are initially found clustered around the sperm DNA (meiosis I and II). However, during mitosis, they disperse to random positions in the cytoplasm. All three antibodies (K48, K63 and proteasome) colocalize with MOs in meiotic embryos. However, only K63 continues to colocalize during mitosis and later (middle column).

In order to further characterize the elimination of sperm-derived organelles from the zygote, we performed Mitotracker labeling of male sperm prior to mating with hermaphrodites that express GFP-tagged ubiquitin (GFP::Ub) in the germline. *C. elegans* hermaphrodites that have been mated with males will preferentially use male sperm for fertilization. After the matings, embryos were cut out of hermaphrodites and imaged with confocal microscopy. Figure 2 shows embryos at a variety of stages from meiosis to the 8-cell stage. Paternal mitochondria are visible as red structures and ubiquitinated MOs are green. The identification of the green (GFP::Ub) structures as MOs is based on the observation that ubiquitin antibodies colocalize with MO antibodies in early embryos (see Figure 3). Just after fertilization, mitochondria and ubiquitinated MOs are visible in an area surrounding the sperm DNA. The mitochondria and MOs do not colocalize, but rather they cluster together in a region around the sperm DNA until the sperm DNA undergoes decondensation and paternal pronucleus formation. After this, the mitochondria and MOs tend to be dispersed randomly throughout the cytoplasm (Figure 2).

**Figure 4:**
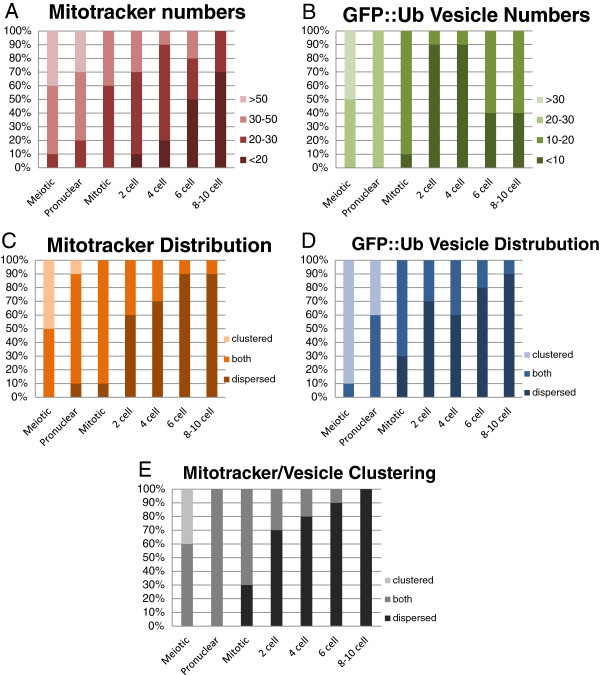
**Tracking sperm organelles after fertilization.** Crosses and microscopy were performed as described in Figure 2. Mitotracker-labeled mitochondria and GFP vesicle counts were taken from maximum projection images. Images were used to determine whether mitochondria or GFP-positive vesicles were clustered (within 2 microns of each other) or dispersed. The stacked bar graphs indicate the percentage of embryos at each stage that showed the phenotype indicated. **A)** Mitochondrial numbers decrease most dramatically between the pronuclear and mitotic phases. **B)** GFP::Ub vesicles (assumed to be MOs) were counted from the same images used for mitochondrial counts. MO numbers decrease with the same kinetics as mitochondria. The slight increase seen in later embryos may indicate vesicles that are not sperm-derived. **C)**. A trend toward dispersion of sperm-derived mitochondria coincides with the decrease in numbers as shown in **A**. **D)** Ubiquitinated MO vesicles also become dispersed as embryos develop. **E)** The clustering of MOs and mitochondria together was assessed. Initially, the mitochondria and MOs cluster together. Following the first mitosis, these two organelles are no longer clustered together. For each trait, ten embryos of each stage were analyzed.

The number of paternal mitochondria was determined by counting Mitotracker labeled structures in Z stacks collected using the confocal microscope. The results of those counts are shown in Figure 4. The data show that the most dramatic change in mitochondria numbers occurs prior to first mitosis (Figure 4A). This coincides with the stage at which the mitochondria become predominantly dispersed rather than clustered together (Figure 4C). This may indicate that mitochondria that are clustered together have an increased likelihood of elimination via autophagy. The GFP::Ub labeled MOs are similar to mitochondria in that the number of MOs show a sharp decline prior to first mitosis (Figure 4B) and the distribution switches to primarily dispersed beginning at the 2-cell stage (Figure 4D). We also examined whether mitochondria and GFP::Ub vesicles cluster together and how that changes over time. Figure 4E shows that clustering of the two organelles is common in newly fertilized embryos, but they show a more dispersed distribution at the end of the meiotic divisions. This change in distribution correlates with the timing of cytoplasmic streaming that occurs at the conclusion of meiosis [20]. It is possible that cytoplasmic streaming is responsible for disrupting the clustering of MOs and paternal mitochondria.

Even though sperm-derived mitochondria undergo a reduction in number soon after fertilization, there are still mitochondria detected even in multi-cellular embryos (Figure 4A). Our results do not explain how these remaining mitochondria might be eliminated. See the discussion for further thoughts on possible mechanisms for this.

### K48-linked and K63-linked ubiquitin chains show different temporal profiles

Ubiquitination of the sperm-derived MOs occurs within minutes after fertilization [1]. Since it is known that the type of ubiquitination can vary, we were interested in pursuing the nature of ubiquitation associated with MOs. Polyubiquitin chains form via linkage to one of each of seven lysines in the ubiquitin protein. Antibodies exist that can specifically recognize the two most prevalent polyubiquitin chains; lysine 48 (K48) and lysine 63 (K63) [21]. Previously, we have shown that both types of linkages can be found on MOs after fertilization [1]. A more detailed analysis of these antibody staining patterns shows that although both chain types are observed, their timing is different.

The chain-specific antibodies were used to stain embryos in conjunction with the 1CB4 antibody that specifically recognizes the MO (Figure 3). Table 1 shows a quantification of these results. The status of the maternal and paternal DNA can be used to determine the stage of the embryo. Figure 3 shows that K48 chains are associated with MOs soon after fertilization, when the maternal DNA is engaged in meiosis I. However, staining for K48 chains fades in meiosis II and is no longer detectable after mitosis begins. In contrast, K63 chains are faint in meiosis I embryos, but become much stronger in meiosis II and later. Antibodies to proteasomes show a temporal profile that is similar to K48 antibody (Figure 3). The similar timing of K48 ubiquitin chains and proteasomes is consistent with the evidence that K48 polyubiquitin chains can serve to target proteins to the proteasome for degradation [6].

**Table 1:** Ubiquitin and proteasome antibody colocalization to MOs in early embryos

	**K48 +**	**K48 -**	**K63 +**	**K63 -**	**Prot +**	**Prot -**
**Meiosis I**	13	3	9	2	10	1
**Meiosis II**	4	3	10	0	0	7
**1-cell**	0	17	22	0	0	9
**2 to 8-cell**	0	14	11	6	0	3

Number indicates the number of embryos showing positive (+) or no (−) colocalization with the MO marker 1CB4.

Interestingly, K63 chains, but not K48 chains also localize to cortical vesicles that appear during meiosis II (Figure 3). There structures are referred to as vesicles because they co-stain with WGA-FITC (L.B. unpublished data). The nature and function of these vesicles is unknown. They are observed only during metaphase of meiosis II and are also observed in strains with germline expressed GFP::ubiquitin.

### Sperm proteasomes do not colocalize with MOs prior to fertilization

One interesting observation from the antibody stains was that sperm stain heavily with the proteasome antibodies, but at very low levels with the anti-ubiquitin antibodies. This is intriguing. Since sperm typically lack the ability to synthesize new proteins [16], it is not clear why sperm would maintain a capacity to degrade proteins. In sea urchins and ascidians, proteasomes released during the acrosome reaction are involved in the penetration of the vitelline envelope by sperm [22,23]. Proteasomes are also found in mammalian acrosomes and it has been proposed that they may serve as a lysin for the zona pellucida [24]. Although a vitelline envelope for *C. elegans* oocytes has not been described, there may be some type of coating that requires a lysin activity [25].

**Figure 5:**
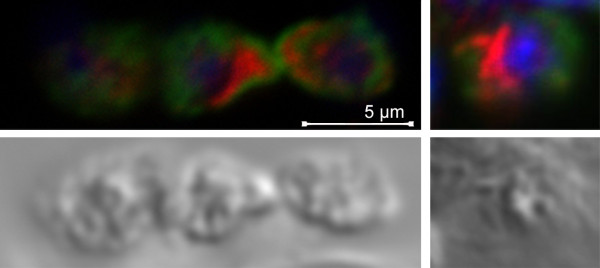
**Spermatozoa contain proteasomes.** Proteasome antibody (red), 1CB4 (green), and DAPI (blue) were used to stain mature sperm from *C. elegans* hermaphrodites. DIC images are shown on the bottom. Three sperm are in the left panel and a single sperm is shown in the right panel. Proteasomes and MOs (1CB4) show minimal overlap. Proteasomes are often found localized to a region between the cell body and the pseudopod (see sperm on right).

Some of the MOs fuse with the plasma membrane in response to sperm activation. In this respect, they share an attribute with the acrosomal vesicle in other species where acrosomal proteasomes are proposed to function as lysins. However, in *C. elegans*, sperm proteasomes do not colocalize with MOs (Figure 5). In fact, proteasomes in sperm tend to be concentrated in a region between the cell body and the pseudopod (Figure 5). How then, do proteasomes become colocalized with MOs after fertilization? It is possible that the proteasomes that localize to MOs after fertilization are maternally derived proteasomes. Another possibility is that the ubiquitination of MO proteins after fertilization causes the proteasomes to localize to MOs.

### Ooctye and sperm proteasomes have different localization patterns after fertilization

**Figure 6:**
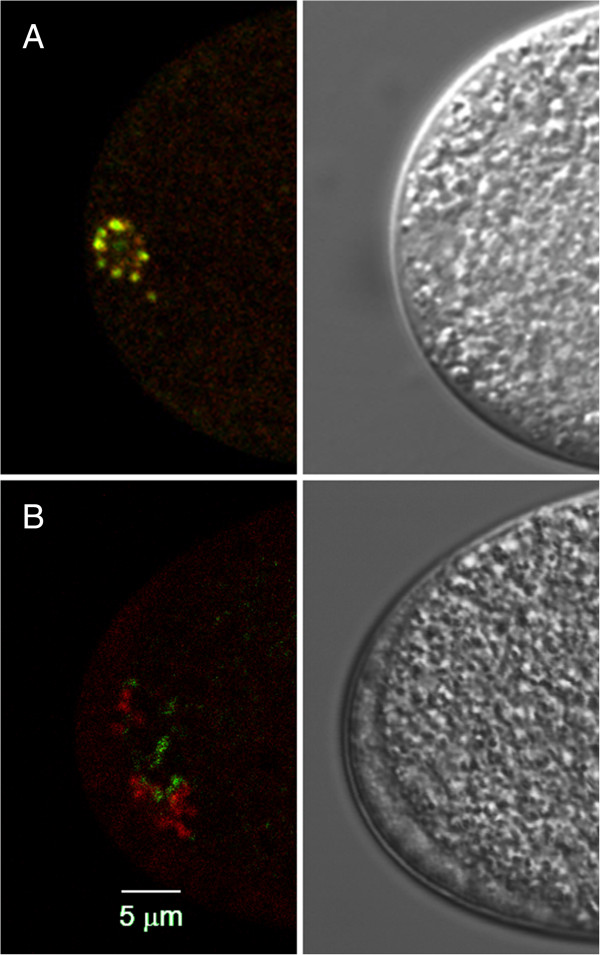
**Paternal and maternal proteasomes have non-overlapping distributions. A)** A newly fertilized embryo with both GFP::Ub (green) and RPT-1::mCherry (red). Colocalization of the two proteins is indicated by the presence of yellow color. This embryo is undergoing maternal meiosis I division at the opposite end (not shown). **B)** Hermaphrodites expressing RPT-1::mCherry were mated to males expressing RPT-1::GFP. The maternal proteasomes (red) localize to structures that resemble the sperm MOs. Wherease, GFP labeled paternal proteasomes (green) are seen in smaller structures near to the maternal proteasomes. This labeling is only seen in meiosis I embryos, not in later embryos.

In order to further investigate the role of sperm-derived proteasomes, transgenic strains were generated that express the proteasomal 19S subunit RPT-1 fused to fluorescent proteins. Two transgenic strains were made: one expressing RPT-1::GFP and another expressing RPT-1::mCherry. The *mex-5* promoter was used to achieve expression in the germline. In both strains, fluorescent transgene expression is detected in the germline in males and hermaphrodites. In fertilized embryos, punctate structures surrounding the sperm DNA appear after fertilization consistent with localization to MOs (Figure 6). RPT-1::mCherry colocalizes with GFP::Ub in newly fertilized embryos indicating that the tagged RPT-1 localizes to the MOs after fertilization (Figure 6A). The RPT-1 fluorescence goes away rapidly and is not detected in pronuclear and mitotic embryos. This temporal pattern is consistent with proteasome and K48 ubiquitin antibody staining (Figure 3 and Table 1).

In *C. elegans,* hermaphroditic reproduction is the norm, however males arise within the population at a low frequency. Males can deliver sperm to hermaphrodites resulting in cross progeny. Embryos generated by crosses between strains harboring different fluorescent markers allows the tracking of sperm-derived materials in the fertilized embryo. By mating the two strains expressing RPT-1 fluorescent proteins, we are able to determine whether paternal or maternal proteasomes localize to the MOs after fertilization. Figure 6B shows an embryo resulting from a cross between males expressing RPT-1::GFP and hermaphrodites expressing RPT-1::mCherry. The fluorescently tagged proteasome from both paternal and maternal proteasomes are present near the sperm DNA. However, the distribution of the two proteasome populations is different and there is very little overlap between the paternal and maternal proteasomes (Figure 6B). Maternal proteasomes exhibit a pattern that is similar to that seen with 1CB4 and ubiquitin, localizing to spherical structures that cluster around the sperm DNA. Whereas the paternal chromosomes localize to the periphery of these structures. When males expressing RPT-1::mCherry are mated to GFP::Ub hermaphrodites, the paternal proteasomes show minimal overlap with the GFP::Ub in fertilized embryos (data not shown). These data indicate that paternal and maternal proteasomes localize to different structures in the fertilized egg and are likely to have different roles in post-fertilization processing of sperm proteins.

## Discussion

### Dual roles for ubiquitination of sperm organelles after fertilization

Fertilization involves the fusion of two gametes to create the zygote. Some cellular material from the sperm is initially present in the zygote but is subsequently eliminated. Data presented in this paper indicates that ubiquitination and proteasomal degradation plays a role in this elimination in *C. elegans.* For convenience the colocalization of ubiquitin chains with the MOs are referred to as “ubiquitinated sperm organelles”. However, it is acknowledged that the target of ubiquitination is most likely a protein (or proteins). Currently, the identity of the protein targets of this ubiquitination is not known. The observation that two types of ubiquitin chains are found associated with sperm organelles after fertilization suggests that there are at least two ubiquitin-related pathways that participate in the processing of these organelles and their associated proteins. The presence of K48 ubiquitin chains on a sperm-derived organelle suggests that a membrane associated sperm protein might be targeted to the proteasome. PEEL-1 is a sperm expressed protein that is toxic unless it is degraded in the embryo [26]. PEEL-1 is a membrane associated protein and may be one of the targets of ubiquitination. It would be interesting to know the identity of any other sperm proteins that are targeted for degradation and the consequences of not degrading them.

K63-linked ubiquitin chains have a substantially different conformation than K48-linked chains [9]. K63 chains have been associated with protein aggregation, signal transduction pathways, and autophagy. Since autophagy is implicated in the clearance of sperm organelles, it is likely that the K63 chains observed here serve as a signal for that process. The K63 chains appear to remain on the MOs until they are eliminated since MO and K63 antibodies continue to colocalize throughout the time period examined. In other systems where ubiquitination serves as a signal for autophagy, proteins have been identified that link these two processes via the pairing of a ubiquitin binding domain (UBA) and a LC3 interacting region (LIR). NBR1 and p62 are two proteins with this domain structure that are known to promote the autophagy of K63 ubiquitinated structures [10]. Although there are no clear homologs for p62 or NBR1 in *C. elegans*, there are proteins that do have a similar domain structure. It will be interesting to see if any of these proteins are required for the elimination of sperm organelles in the zygote.

Since the mitochondria themselves are not ubiquitinated, they are thought to be swept up into autophagosomes via their close association with ubiquitinated MOs. Interestingly, the association between MOs and mitochondria changes as the oocyte completes meiosis and transitions to a zygote. This clustering of the two organelles is thought to be important for the autophagic removal of mitochondria [27]. However, after the pronuclei have formed and the embryo enters into mitotic cell divisions, the mitochondria and MOs are no longer found in clusters. Therefore, it is not known how the remaining sperm mitochondria are eliminated. It is possible that mitochondrial morphology may play a role in the removal. Sperm mitochondria are small and relatively spherical. Whereas, oocyte mitochondria are predominantly elongated and reticular/tubular [28] (CH and LB unpublished data). It has been proposed that smaller mitochondria are more susceptible to mitophagy [29]. Thus the morphology of sperm mitochondria may contribute to their elimination from the embryo.

### The function of sperm proteasomes still to be determined

In sea urchins and mammals, it has been proposed that sperm secrete proteasomes that function as the proteolytic mechanism for breaking through the vitelline envelope [30]. It is not clear whether proteasomes from *C. elegans* sperm have a similar function. Our localization analysis indicates that proteasomes are cytosolic in *C. elegans* sperm and not associated with an acrosome-like structure. Furthermore, some sperm proteasomes are internalized at fertilization. Further studies are needed in order to investigate the role of sperm proteasomes in the process of fertilization in this system.

## Conclusions

Two different types of polyubiquitin chains along with proteasomes are associated with sperm organelles after fertilization in *C. elegans* embryos. K48 ubiquitin chains and proteasomes are present for a brief time. Whereas, K63 chains persist on sperm organelles through several cell cycles and could potentially contribute to autophagy of paternal organelles.

## Methods

### Worm strains and maintenance

Worms were maintained at 20°C on nematode growth media with OP50 *E. coli* according to standard methods [31]. The wild type Bristol N2 nematode strain and the OP50 bacterial strain were obtained from the Caenorhabditis Genetics Center. The LN129 strain [*Ppie-1::GFP::ubiquitin + unc-119(+); unc-119(ed3)*] was obtained by biolistic transformation (Bio-Rad PDS-1000) of pLB3 plasmid (pID2.02 vector with GFP::ubiquitin insert) into the HT1593 nematode strain. Transformants were selected by starvation at 25°C for 2 weeks. The resulting strain was crossed to OCF1 carrying the mCherry::histone transgene (*itIs37*) creating the LN130 strain [*Ppie-1::GFP::ubiquitin + unc-119(+); Ppie-1::mCherry::his-58*].

The LN151 [P*mex-5*::*rpt-1*::*mCherry* + *unc-119*^*+*^ II; *unc-119 (ed3)*] and LN152 [P*mex-5*::*rpt-1*::*GFP* + *unc-119*^*+*^ II; *unc-119 (ed3)*] strains were generated by MoSci site specific integration [32]. Plasmids for MoSci injection were created using Gateway recombination of the *mex-5* promoter, the *rpt-1* ORF (OpenBiosystems), and either mCherry or GFP ORF with *tbb-2* 3-UTR into the vector pCFJ150 which contains flanking Mos sites. The resulting plasmids (55 ng/μl) were co-injected into the EG6699 strain along with pMA122 (15 ng/μl), pCFJ601 (50 ng/μl), and pLB10 (10 ng/μl). Injected worms were grown at 25°C for 4 days and non-Unc F1 progeny were screened for germline fluorescence using a fluorescent dissecting microscope (Tritech Research).

### Male populations and mating

Male N2, LN130, LN151, and LN152 populations were generated by treatment of 20 L4 hermaphrodites in 7% ethanol in M9 buffer at room temperature for 20 minutes. N2 and LN130 male populations were maintained by mating 7–10 males with 3–6 hermaphrodites on 60 mm NGM plates seeded with 100 μL saturated overnight OP50 *E. coli* culture. Matings between strains were performed by placing 10 hermaphrodites and 10–20 males on a seeded NGM plate at 20°C overnight. Plates were transferred to 25°C a few hours before observation.

### Antibodies

Primary antibodies used were rabbit anti-K63 ubiquitin (Apu3 from Millipore), rabbit anti-K48 ubiquitin (Apu2 from Millipore) and mouse monoclonal anti-1CB4 (generous gift from Steve L’Hernault at Emory University). Secondary antibodies used in immunofluorescence were goat-anti-mouse FITC and goat-anti-rabbit TRITC (Jackson ImmunoResearch Laboratories).

### Antibody staining

Colocalization of K48-linked ubiquitin, K63-linked ubiquitin and 1CB4 was determined using K48 and K63 specific antibodies (1:100) with 1CB4 (1:10). Gravid adults were cut open on poly-L-lysine-coated slides to release embryos. Slides were placed in liquid nitrogen for 5 minutes and then fixed with −20° methanol for 20 minutes. Slides were incubated in primary antibody at 4° overnight followed by incubation with secondary antibody (1:200) for 2 hours at room temperature. Antibodies were diluted in PBST:30% NGS (normal goat serum) and washes were done in PBST (PBS with 0.5% Tween-20). Vectasheild plus DAPI (Vector Labs) was used for mounting. Control stains lacking linkage-specific primary antibody showed no rhodamine fluorescence colocalizing with 1CB4. Embryos were observed with a Zeiss LSM 700 confocal microscope using Zen 2009 software.

### MitoTracker staining and fluorescent protein tracking

N2 male sperm were labeled with MitoTracker Red CMXRos (Invitrogen) by modification of a protocol described previously [33]. MitoTracker was dissolved in DMSO to a concentration of 1 mM. Forty to fifty late L4/young adult N2 males were washed and transferred to 300 μL of 10 μM MitoTracker in M9 solution. Males were incubated with MitoTracker for two hours at 25°C. Males were then washed with M9 buffer and split onto two 60 mm NGM plates seeded with 100 μL saturated overnight OP50 *E. coli* culture. Six to eight L4 LN130 hermaphrodites were added to each NGM plate. N2 males and LN130 hermaphrodites were allowed to mate overnight at 25°C. Observation of embryos was performed as described in Boyd *et al*. [34] using a Zeiss LSM 700 confocal microscope with Zen 2009 software.

LN151 X LN152 and LN151 X LN130 cross embryos were observed on a Nikon C1 confocal microscope.

## Competing interests

The authors declare that they have no competing interests.

## Authors’ contributions

CH performed the mitochondrial tracking experiments, wrote portions of the manuscript, and created Figures 2 and 4. KMH performed crosses for the fluorescent proteasome strains. LB performed the antibody stains, created the transgenic strains, and wrote the manuscript. All authors read and approved the article.
